# Long-Term Results of a Phase 2 Study of Definitive Chemoradiation Therapy Using S-1 for Esophageal Squamous Cell Carcinoma Patients Who Were Elderly or With Serious Comorbidities

**DOI:** 10.3389/fonc.2022.839765

**Published:** 2022-04-05

**Authors:** Yun Chen, Zhengfei Zhu, Weixin Zhao, Qi Liu, Junhua Zhang, Jiaying Deng, Dashan Ai, Saiquan Lu, Liuqing Jiang, Ihsuan Tseng, Huixun Jia, Kuaile Zhao

**Affiliations:** ^1^Department of Radiation Oncology, Fudan University Shanghai Cancer Center, Shanghai, China; ^2^Department of Oncology, Shanghai Medical College, Fudan University, Shanghai, China; ^3^Shanghai Key Laboratory of Radiation Oncology, Shanghai, China; ^4^Department of Radiation Oncology, Fujian Medical University Union Hospital, Fuzhou, China; ^5^Clinical Statistics Center, Fudan University Shanghai Cancer Center, Shanghai, China; ^6^Department of Ophthalmology, Shanghai General Hospital, Shanghai, China

**Keywords:** esophageal squamous cell carcinoma, S-1, definitive chemoradiotherapy, elderly, serious comorbidities

## Abstract

**Background:**

The optimal evidence-based management for the subsets of locally advanced esophageal squamous cell carcinoma (ESCC) patients who rejected or were intolerant to intravenous chemotherapy due to old age or serious comorbidities is currently lacking. This study aimed to assess the safety and local control rate (LCR) of S-1 (tegafur–gimeracil–oteracil potassium) combined with radiotherapy in these subsets of ESCC patients.

**Methods:**

Locally advanced ESCC patients who rejected or were intolerant to intravenous chemotherapy due to age >75 years or serious comorbidities were enrolled in a prospective, single-arm, phase 2 trial. The patients were treated with definitive concurrent chemoradiotherapy with S-1, which was administered orally twice daily for 28 days. The radiotherapy dose was 61.2 Gy delivered in 34 fractions. The primary end-point was the 3-year LCR.

**Results:**

One hundred five ESCC patients were recruited between March 2013 and October 2015. At the median follow-up of 73.1 months (IQR 65.5–81.4 months), 3-year LCR was 61.1%, and 1, 3, and 5-year overall survival was 77.9, 42.3, and 24.8% respectively. For safety analysis, ≥grade 3 acute adverse events included thrombocytopenia (6.7%), leukopenia (2.9%), anemia (1.0%), anorexia (1.0%), fatigue (10.5%), hiccup (1.0%), pneumonitis (4.8%), and esophagitis (3.8%). Two patients (1.9%) died of late esophageal hemorrhage, and one patient (1.0%) died of late radiation-induced pneumonitis.

**Conclusion:**

S-1 is a promising regimen in concurrent chemoradiotherapy with low toxicity and a favorable LCR in ESCC patients who rejected or were intolerant to intravenous chemotherapy due to old age or serious comorbidities.

**Clinical Trial Registration:**

ClinicalTrials.gov, NCT01831531.

## Introduction

Esophageal cancer (EC) is the 4th most common cause of cancer deaths in China (1). The RTOG85-01 trial established the efficacy of definitive concurrent chemoradiotherapy (dCRT), which can significantly improve survival compared with radiotherapy (RT) alone (2). All patients in the RT alone arm died of cancer by 3 years. Since then, dCRT has become the standard treatment for inoperable locally advanced EC patients, and RT alone should only be reserved for palliation or for patients who are medically unable to receive chemotherapy. However, there is indeed a group of EC patients who rejected or were intolerant to intravenous chemotherapy due to old age or serious comorbidities. The management of these patients is a therapeutic challenge. Searching an alternative effective chemotherapy agent with moderate treatment related toxicities seems to be a promising strategy for these patients.

S-1 is an oral chemotherapy agent of fluoropyrimidine, consisting of tegafur, gimeracil, and oteracil potassium, and has been proven to be noninferior in efficacy to infusional fluorouracil in gastric cancer (3). In addition, the S-1-based regimen showed a good safety profile with lower incidence of grade 3/4 neutropenia (OR = 0.33) than the 5-fluorouracil based regimen in advanced gastric cancer (4). Moderate toxicities and promising response rates were also observed in EC patients treated with concurrent chemoradiotherapy with an S-1 based regimen (5, 6). Therefore, S-1 combined with definitive RT may be an optimal option for locally advanced EC patients who rejected or were intolerant to intravenous chemotherapy due to old age or serious comorbidities.

In this phase 2 clinical trial (ESO-Shanghai 7), we aimed to verify the safety and efficacy of definitive RT combined with S-1 alone in locally advanced esophageal squamous cell carcinoma (ESCC) patients who rejected or were intolerant to intravenous chemotherapy due to old age or serious comorbidities. We hypothesized that S-1 combined with radiotherapy had low toxicity and improves local control in these ESCC patients.

## Materials and Methods

### Study Design

This single arm phase 2 clinical trial was performed at the Fudan University Shanghai Cancer Center (FUSCC). Eligible patients were histologically confirmed diagnosis of ESCC, stage IIa to IVa, and were previously untreated. They rejected or were intolerant to intravenous chemotherapy due to old age (more than 75 years), or serious comorbidities (namely, severe cardiovascular diseases, sequelae of cerebral infarction, uncontrolled diabetes, etc.). Other inclusion criteria were an Eastern Cooperative Oncology Group (ECOG) performance status of 0 to 2, a life expectancy of at least 3 months, adequate organ function (hemoglobin ≥9 g/dl, white blood count ≥3 × 10^9^/L, neutrophil count ≥1.5 × 10^9^/L, platelet count ≥ 70 × 10^9^/L, aspartate transaminase and alanine aminotransferase <2.5 × upper limit of normal (ULN), total bilirubin <1.5× ULN, and creatinine <1.5× ULN), and the use of an effective contraceptive for adults to prevent pregnancy. The major exclusion criteria were: complete esophageal obstruction, distant metastatic disease, drug addiction, alcoholism or AIDS, and other concomitant cancers. For the full inclusion and exclusion criteria, see **Appendix 1**.

### Treatment

S-1 was given orally as described from days 1 to 28 at the beginning of the first fraction of radiotherapy. The dose of S-1 was calculated according to body surface area (BSA) (<1.6 m^2^: 40 mg bid and ≥1.6 m^2^: 50 mg bid). A powder form of S-1 would be administered if patients could not swallow the oral capsule. If patients had hematologic toxic effects of grade 4 or nonhematologic toxic effects of more than grade 3, their daily dose was reduced, from 100 to 80 mg or from 80 to 60 mg.

A total dose of 61.2 Gy was prescribed at the isocenter delivered by 6 MV photons in 34 fractions of 1.8 Gy (five fractions per week, one fraction per day). Intensity modulated radiotherapy based on a CT simulation planning system with a 5 mm thickness scan slice throughout the entire neck and thorax was required. Involved-field irradiation was used in this study. The gross tumor volume (GTV) was defined as visible esophageal tumor and metastatic lymph nodes based on the imaging of endoscopic ultrasound, esophageal radiography, or CT scan (whichever was larger). The criteria for metastatic lymph nodes were as follows: pathologic confirmation or short axis of ≥10 mm in the mediastinum or cervix, or short axis of ≥5 mm in the tracheoesophageal groove, or histologically proven as metastatic by puncture. The clinical target volume (CTV) included the GTV manually extended by 30 mm in the superior–inferior direction for potential submucosal invasions. Metastasis lymph nodes had no CTV. A further 1 cm expansion added to the CTV in all directions was applied to account for technical uncertainties, which defined the planning target volume (PTV). The field next to the spinal cord could be slightly changed in order to reduce the exposure of the spinal cord. The criteria of dose distribution were as follows: 95% of the PTV to receive ≥99% of the prescribed dose, 99% of the PTV to receive ≥95% of the prescribed dose, <2cm^3^ of the PTV to receive ≥120% of the prescribed dose, and <1 cm^3^ of the PTV to receive ≥110% of the prescribed dose. Highest and lowest point dose inside PTV were recorded. Normal organ dose restrictions were defined as follows: spinal cord: the highest point dose has to be less than 45 Gy; lung: The volume of lung (PTV excluded) receiving 20 Gy has to be equal to or less than 30% of the total lung volume, and the mean lung dose has to be equal to or less than 15 Gy at the same time; and heart: the mean dose has to be less than 40 Gy.

### Outcomes

The primary endpoints of this trial included the 3-year local control rate (included the primary esophageal tumor and regional lymph node failure) and the number and grade of participants with adverse events (AEs). The secondary endpoint was the overall survival (OS). We defined OS as the time between the start of the study treatment (Day 1) and death from any cause or the last follow-up for patients alive at the end of the study. We defined locoregional failure as either failure within the primary esophageal tumor or the area of regional lymph node and distant failure as failure in distant organ or non-regional lymph node area.

### Statistical Analysis

As a retrospective study done by our team showing a 3-year local control rate of 46% in ESCC patients aged ≥70 years treated with radiotherapy alone (7), we designed this phase 2 study to see if radiotherapy concurrent with S-1 regimen could achieve a 3-year local control rate of 60%. Assuming a type I error rate of 0.05 and a type II error rate of 0.20, 103 assessable patients were needed to provide a statistically significant difference between 46 and 60% with 80% power. Adjusting this figure by 2% to account for patient ineligibility or loss, a total sample size of 105 will be needed for the study. We used the Kaplan–Meier method and log-rank tests to estimate the local control rate and OS. Cox regression was used to estimate the hazard ratios. Data were analyzed with SPSS version 19.0 (SPSS Inc., Chicago, IL, USA).

## Results

**Figure 1:**
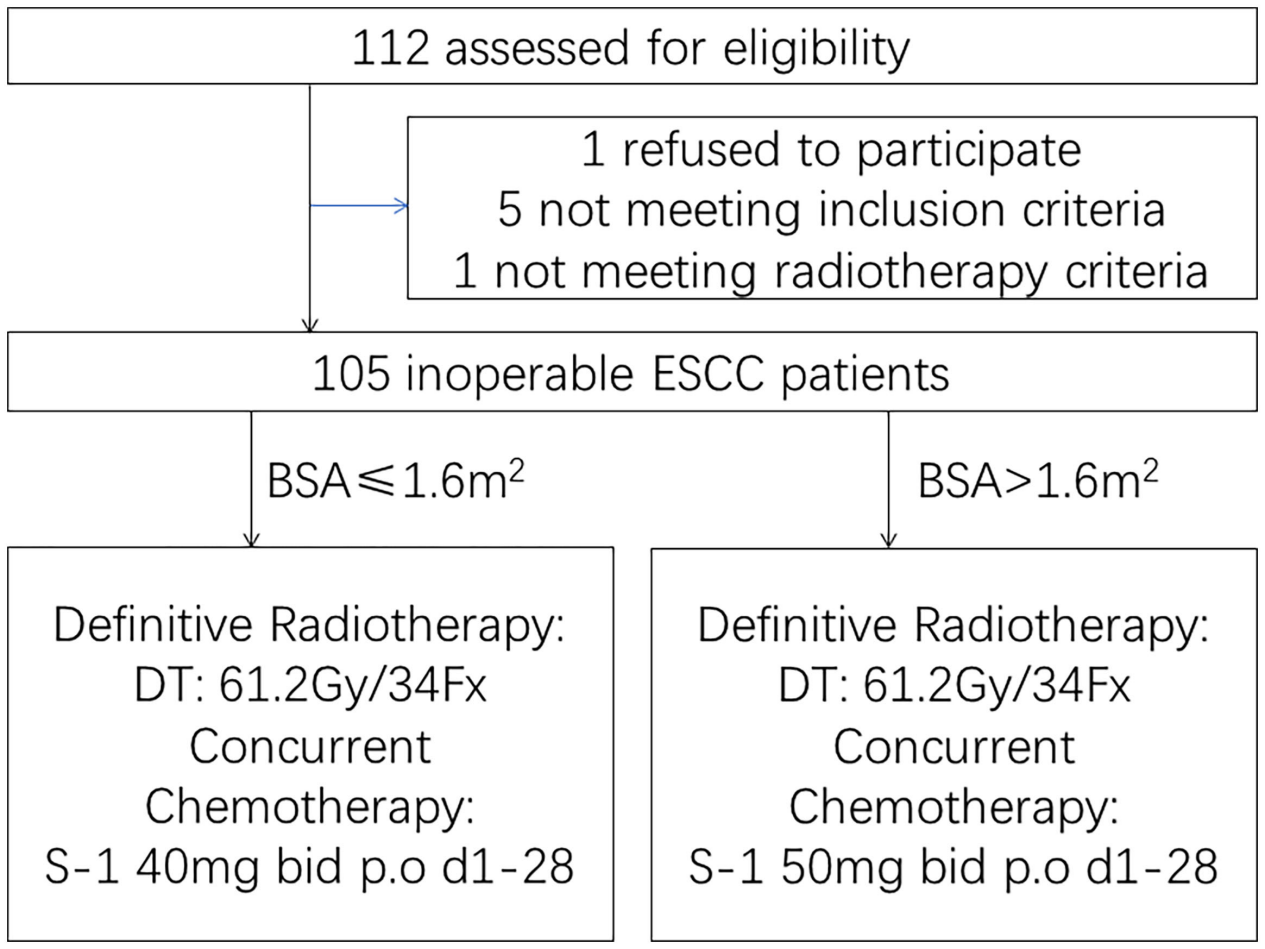
Trial profile. One hundred five inoperable esophageal squamous cell carcinoma patients who were aged >75 years or rejected or were intolerant to intravenous chemotherapy due to serious medical comorbidities were enrolled.

From March 2013 to October 2015, a total of 105 patients with ESCC were enrolled in the Fudan University Shanghai Cancer Center (**Figure 1**). The baseline and tumor characteristics of the enrolled patients are listed in **Table 1**. The median age was 77 years (IQR 72–80). Seven (6.7%) patients who were initially staged as stage IVa were upstaged to IVb due to the incorrect staging for supraclavicular lymph node metastasis after staging review. Two patients were initially staged as stage IIa were downstage to I due to reevaluation by endoscopic ultrasound. The median tumor lengths of the patients enrolled were 5.3 cm (IQR 3.8–6.5). Details of serious comorbidities of patients enrolled are listed in **Table A.1**.

**Table 1:** Characteristic parameters of enrolled patients.

Characteristics	No. of patients (N = 105, %)
Sex	
Male	81 (77.1)
Female	24 (22.9)
Age, years	
>85	3 (2.9)
81–85	19 (18.1)
76–80	41 (39.0)
71–75	19 (18.1)
≤70	23 (21.9)
Smoking history	
Never	45 (42.9)
Former or current	60 (57.1)
Drinking history	
Never	55 (52.4)
Former or current	50 (47.6)
Stage (AJCC 6th)	
I*	2 (1.9)
IIa	34 (32.4)
IIb	10 (9.5)
III	47 (44.8)
IVa	5 (4.8)
IVb*	7 (6.7)
Site	
Cervical	7 (6.7)
Upper	28 (26.7)
Middle	61 (58.1)
Lower	9 (8.6)
Tumor length, cm^#^	5.3 ± 2.2
≤7	88 (83.5)
>7	17 (16.2)
ECOG	
0	29 (27.6)
1	51 (48.6)
2	25 (23.8)
BSA	
≤1.6 m^2^	28 (26.7)
>1.6 m^2^	77 (73.3)
Subgroups of patients enrolled	
Aged >75 years without serious comorbidities	47 (44.8)
Aged >75 years with serious comorbidities	16 (15.2)
Aged ≤75 with serious comorbidities	35 (33.3)
Aged ≤75 refusal	7 (6.7)

AJCC, American Joint Committee on Cancer; ECOG, Eastern Cooperative Oncology Group performance status; BSA, body surface area.

^*^ Two patients had incorrect T staging and the stage was changed from IIa to I after reevaluation by endoscopic ultrasound. Seven patients had incorrect staging for supraclavicular lymph node metastasis, and the stage was changed from IVa to IVb after staging review.

^#^Data are mean ± SD with available data.

Sixty-eight (64.8%) patients completed the full treatment, namely, 93 (88.6%) patients completed the full radiotherapy prescribed and 70 (66.7%) patients completed the full-prescribed dose of S-1. The details of the treatment compliance are shown in **Table A.2**. Treatment delay and cessation were mainly due to treatment-induced toxicities. One hundred one (96.2%a patients received at least 50 Gy radiotherapy.

**Figure 2:**
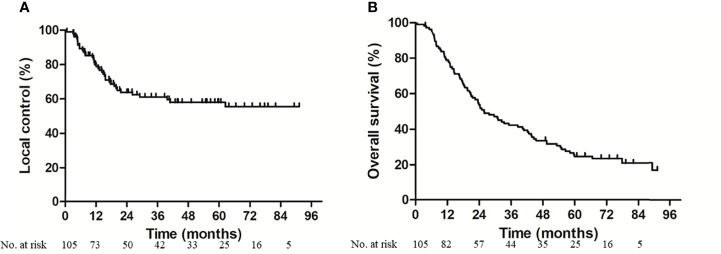
Local control **(A)** and overall survival **(B)** for enrolled patients. At the median follow-up of 73.1 months, 3-year local control rate and overall survival was 61.1 and 42.3% respectively.

At the data cutoff date (October 31, 2020), the median follow-up of the surviving patients was 73.1 months (IQR 65.5–81.4 months). Two patients were lost to follow-up. Twenty-three (21.9%) patients were alive without local disease progression. The 1, 3, and 5-year local control rates were 77.8, 61.1, and 58.1%, respectively (**Figure 2A**). Twenty-three (21.9%) were alive without metastasis. The patterns of treatment failure are shown in **Table 2**. Eighty-one (77.1%) patients suffered deaths at the time of analysis, namely, 59 patients who died of tumor progression and 22 patients who died of other causes. The median survival was 26.1 months. The 1, 3, and 5-year OS rates were 77.9, 42.3, and 24.8%, respectively (**Figure 2B**). Moreover, the differences in terms of OS between the patients aged >75 years and patients aged ≤75 years, and between the patients with serious comorbidities and patients without serious comorbidities were not significant (**Figure A.1****)**.

**Table 2:** Pattern of treatment failure.

Characteristics	No. of patients (N = 105, %)
Live without treatment failure	22 (21.0)
Failure	83 (79.0)
– Locoregional only	29 (27.6)
− Distant only	22 (21.0)
− Locoregional and distant	10 (9.5)
− Died of other cause	22 (21.0)
− Second primary tumor	8 (7.6)
− Toxicity-induced death	3 (2.9)
− Comorbidities	11 (10.5)
Locoregional failure time	
− Within 1 year	20 (19.0)
− Within 2 years	14 (13.3)
− Within 3 years	5 (4.8)
Locoregional failure subgroup	
Tumor stage	
− I–II	15/46 (14.3)
− III–IV	24/59 (40.7)
Age	
− >75	27/63 (42.9)
− ≤75	12/42 (28.6)

Since all patients received at least one dose of S-1, the safety population was equal to the intention-to-treat population. All ≥grade 2 side effects and grade 1 side effects that occurred in more than 10% of the patients reported during treatment are shown in **Table 3**. Seventy (66.7%) patients had ≥grade 2 acute side effects from the treatment, most of which were related to leukopenia and radiation-induced esophagitis. Twenty-six (24.8%) patients had grade 3 or above adverse events, namely, anemia, leukopenia, thrombocytopenia, anorexia, hiccups, fatigue, radiation-induced pneumonitis, and esophagitis. Two patients (1.9%) died of late esophageal hemorrhage, and one patient (1.0%) died of late radiation-induced pneumonitis.

**Table 3:** Side effects of patients enrolled.

Side effects*	No. of patients (N = 105, %)					
	Grade 1		Grade 2	Grade 3	Grade 4	Grade 5
**Acute side effects**
Anemia	54 (51.4)	2 (1.9)		1 (1.0)	0	0
Leukopenia	43 (41.0)	26 (24.8)		2 (1.9)	1 (1.0)	–
Thrombocytopenia	27 (25.7)	10 (9.5)		6 (5.7)	1 (1.0)	–
Anorexia	27 (25.7)	7 (6.7)		1 (1.0)	0	0
Nausea	17 (16.2)	11 (10.5)		0	–	–
Vomiting	6 (5.7)	5 (4.8)		0	0	0
Fatigue	24 (22.9)	9 (8.6)		11 (10.5)	–	–
Fever	6 (5.7)	3 (2.9)		0	0	0
Hiccups	5 (4.8)	0		1 (1.0)	–	–
Cardiac disorders	8 (7.6)	0		0	0	0
Radiation-induced dermatitis	6 (5.7)	1 (1.0)		0	0	0
Radiation-induced esophagitis	57 (54.3)	17 (16.2)		4 (3.8)	0	0
Radiation-induced pneumonitis	33 (31.4)	19 (18.1)		4 (3.8)	1 (1.0)	0
**Late side effects**						
Cardiac	0	0		0	0	0
Radiation-induced esophagitis	1 (1.0)	5 (4.8)		0 (0.0)	0 (0.0)	2 (1.9)^#^
Radiation-induced pneumonitis	18 (17.1)	0 (0.0)		0 (0.0)	0 (0.0)	1 (1.0)

^*^All ≥Grade 2 side effects and Grade 1 side effects that occurred in more than 10% of patients reported during treatment.

^#^Patients died of esophageal hemorrhage without clear evidence of progression.

## Discussion

Definitive concurrent chemoradiotherapy is the standard treatment for inoperable esophageal cancer patients. The RTOG 85-01 trial showed that EC patients treated with cisplatin plus fluorouracil concurrent with radiotherapy had significantly better overall survival than those treated with radiotherapy alone (2). Likewise, our previous ESO-Shanghai 1 trial, enrolling ESCC patients aged 18–75 without serious medical comorbidities, showed a promising 3-year OS in both dCRT with cisplatin plus fluorouracil (51.8%) and dCRT with paclitaxel plus fluorouracil (55.4%) (8). However, the optimal evidence-based management for the EC patients who rejected or were intolerant to intravenous chemotherapy due to old age or serious comorbidities is currently lacking. The long-term results of this prospective phase 2 trial showed a promising local control and a low incidence of side effects of dCRT with S-1 alone when treating this group of ESCC patients. The 3-year local control rate in this trial was comparable with that of the ESO-Shanghai 1 trial (61% vs. 62.2%) and the incidence of ≥grade 3 acute AEs was much lower (24.8% vs. 50.2%) (8).

With global aging, it is very important to understand the treatment of geriatric cancer patients. However, most of clinical trials excluded elderly patients because of the high risk of treatment-related morbidity and mortality, limited life expectancy, and functional status. The treatment effects of this age group were underrepresentation with paucity of data (9, 10). Chemotherapy concurrently combined with radiotherapy is often considered to be too toxic for most elderly EC patients. Jingu et al. showed concurrent chemotherapy with radiotherapy for esophageal cancer in patients aged 80 years or older did not have a significant OS benefit but led to significantly more severe late toxicities than RT alone (11). Likewise, Xu et al. compared patients ≥80 years with esophageal cancer treated with conventional dCRT with 2 younger patient cohorts (65–79 years and <65 years) treated with dCRT by propensity score matching and showed that the elderly cohort exhibited statistically significant evidence of an increased rate of severe radiation pneumonitis (12). Several studies have suggested that dCRT improves overall survival in elderly patients only in locally advanced stage compared with RT alone (13, 14). Since elderly patients have unique issues, namely, life expectancy, comorbidities, and the risk of treatment-induced morbidity, chemoradiotherapy requires careful consideration and should be carefully selected in elderly EC patients (10).

Single agent or doublet agents, such as docetaxel, nedaplatin/5-fluorouracil, and cisplatin/capecitabine, have been combined with radiotherapy for treating with elderly EC patients in several studies and have achieved promising survival results (15–18). However, for elderly EC patients, compared with double-agent-based dCRT, single-agent-based dCRT was considered to have a lower incidence of treatment side effects and comparable OS (19, 20). The long-term results of a retrospective analysis of elderly ESCC patients treated with S-1 concurrent with radiotherapy showed satisfactory survival outcomes and tolerable toxicities (21). Furthermore, several prospective trials also showed mild toxicity and satisfactory efficacy in elderly ESCC patients treated with S-1 concurrent with radiotherapy (22–24). In our prospective trial, S-1 concurrent with definitive radiotherapy was well tolerated in either elderly patients or patients with serious comorbidities. The incidence of ≥grade 3 toxicities in each side effect was less than 10%, which was comparable to previous studies with single S1 and much lower than that with doublet agents in dCRT in elderly ESCC patients (21–25). The median survival time in present trial was favorable (26.1 months) and comparable to previous similar studies (22.6–25.7 months) (21–24).

A phase 2 trial using a single agent of platinum concurrent with radiotherapy of 50 Gy in 30 elderly EC patients and showed mediocre mid-term efficacy with a 3-year OS of only 22.2%, and nine patients died from local failure. They suggested that the therapeutic ratio or locoregional control might be improved by increasing the radiotherapy dose or by testing new radiosensitizer agents (26). In this phase 2 trial, we used a radiotherapy dosage of 61.2 Gy according to the treatment guidelines of radiotherapy for Chinese esophageal carcinoma and achieved a promising 3-year local control rate (61.1%) and OS (1, 3, and 5-year OS of 77.9, 42.3, and 24.8%, respectively) (27). Our results were comparable to the long-term results of a retrospective study treating elderly ESCC patients with S-1 concurrent with radiation doses of 54.0–60.0 Gy (21). The 1, 3, and 5-year OS in that study were 70.6, 41.8, and 25.9% respectively.

It is known that comorbidities have an independent prognostic effect on cancer patients (28). However, few studies have focused on comorbidities in the treatment decision of EC patients. Patients with serious comorbidities, such as chronic diseases of the cardiovascular, pulmonary, and hepatic systems, are usually excluded from clinical trials. In this phase 2 trial, the enrollment included this group of ESCC patients, and promising local control and good tolerance of S-1 concurrent with definitive RT with low treatment toxicities were observed. However, we did observe that a high percentage of patients (13.3%) died of nononcologic causes, which is undoubtedly related to the aging and serious comorbidities pretreatment.

Our study had several limitations. First, we did not set the control group of radiotherapy alone, and a randomized controlled study would be ideal for the comparison. A randomized phase 3 trial comparing simultaneous integrated boost radiotherapy with or without S-1 is ongoing (29). Another limitation is that we did not assess the quality of life of patients enrolled, which may offer more comprehensive knowledge of the safety results of S-1 concurrent with radiotherapy when treating ESCC patients who were elderly or had serious comorbidities.

## Conclusion

In summary, the long-term results of this phase 2 trial demonstrated that S-1 concurrent with radiotherapy was well tolerated in ESCC patients who rejected or were intolerant to intravenous chemotherapy due to old age or serious comorbidities. The promising 3-year local control rate suggests that this approach was effective and merits randomized phase 3 trial evaluation.

## Author’s Note

Parts of this manuscript were previously presented in 2016 ASTRO meeting and 2019 ASTRO meeting, respectively.

## Data Availability Statement

The original contributions presented in the study are included in the article/**Supplementary Material**. Further inquiries can be directed to the corresponding author.

## Ethics Statement

The studies involving human participants were reviewed and approved by the Ethical Committee of Fudan University Shanghai Cancer Center. The patients/participants provided their written informed consent to participate in this study.

## Funding

This trial was supported by the National Natural Science Foundation of China (grant 81703160).

## Conflict of Interest

The authors declare that the research was conducted in the absence of any commercial or financial relationships that could be construed as a potential conflict of interest.

## Publisher’s Note

All claims expressed in this article are solely those of the authors and do not necessarily represent those of their affiliated organizations, or those of the publisher, the editors and the reviewers. Any product that may be evaluated in this article, or claim that may be made by its manufacturer, is not guaranteed or endorsed by the publisher.

## Supplementary Material

Supplementary Figure 1 Overall survival for subgroups of patients enrolled. No significant difference in overall survival was observed neither between the patients aged >75 years and patients aged ≤75 years **(A)**, nor between the patients with serious comorbidities and patients without serious comorbidities **(B)**.

## Abbreviations

AEs, adverse events; BSA, body surface area; CTV, clinical target volume; dCRT, definitive concurrent chemoradiotherapy; EC, Esophageal cancer; ECOG, Eastern Cooperative Oncology Group; ESCC, esophageal squamous cell carcinoma; FUSCC, Fudan University Shanghai Cancer Center; GTV, gross tumor volume; OS, overall survival; PTV, planning target volume; RT, radiotherapy; ULN, upper limit of normal.
