# Supplementary material for: Long-Term Results of a Phase 2 Study of Definitive Chemoradiation Therapy Using S-1 for Esophageal Squamous Cell Carcinoma Patients Who Were Elderly or With Serious Comorbidities
Source: Front Oncol. 2022 Apr 5;12:839765. doi: 10.3389/fonc.2022.839765 (PMC9016823; doi:10.3389/fonc.2022.839765)
Supplement: Supplementary file 1 [file Table_1.pdf]

Table A.1. List of Serious Comorbidities of Enrolled Patients

Serious comorbidities*	**No. of patients (N=105, %)**
Symptomatic coronary disease	18 (17.1)
Significant ventricular arrhythmia requiring medication	12 (11.4)
Hepatic sclerosis	3 (2.9)
Chronic renal disease requiring medication	4 (3.8)
Chronic bronchitis requiring medication	11 (10.5)
Sequelae of cerebral infarction	9 (8.6)
Uncontrolled diabetes	5 (4.8)
Autoimmune disease requiring medication	5 (4.8)
Metabolic arthritis requiring medication	2 (1.9)
Controlled psychiatric disorders requiring medication	2 (1.9)

* Thirty-nine patients had one kind of serious comorbidity, seven patients had two kinds of serious comorbidities, four patients had three kinds of serious comorbidities, and one patient had four kinds of serious comorbidities.

Table A.2. Treatment compliance and radiotherapy parameters and in patients enrolled

Treatment compliance and radiotherapy parameters	No. of patients (N=105, %)
**Chemotherapy compliance**	
Completed	70 (66.7)
No completed	35 (36.2)
**S-1 Doses received**	
< 25%	6 (5.7)
≥ 25% but < 50%	10 (9.5)
≥ 50% but < 75%	14 (13.3)
≥ 75% but < 100%	5 (4.8)
100%	70 (66.7)
**Reasons for premature cessation of chemotherapy**	
Refusal	8 (7.6)
Treatment-induced toxicities	22 (21.0)
Comorbidity	5 (4.8)
**Chemotherapy delays**	
No	93 (88.6)
Within two weeks	9 (8.6)
More than two weeks	3 (2.9)
**Radiotherapy compliance**	
Completed	93 (88.6)
No completed	12 (11.4)
**Radiotherapy parameters**	
Dose (Gy) #	59.6 ± 5.9
- 61.2Gy	93 (88.6)
- Not completed, but total dose ≥50Gy	8 (7.6)
- Not completed, but total dose <50Gy	4 (3.8)
GTV (cm^3) #	38.2 ± 25.7
≤ 40	66 (62.9)
> 40	39 (37.1)
PTV (cm^3) #	301.4 ± 139.2
Lung V5 (%) #	55.6 ± 11.8
Lung V20 (%) #	20.7 ± 4.9
Mean lung dose (Gy) #	11.7 ± 2.3
Heart V30 (%) #	27.6 ± 17.9
Mean heart dose (Gy) #	18.8 ± 10.5
≤ 10	27 (25.7)
> 10	78 (74.3)

**Reasons for premature cessation of radiotherapy**	
Refusal	3 (2.9)
Treatment-induced toxicities	6 (5.8)
Comorbidity	3 (2.9)
**Deliver over the planned overall radiotherapy time**	
No delay	98 (93.3)
Within two weeks	4 (3.8)
More than two weeks	3 (2.9)

[#] Data are mean ± SD with available data
