# Supplementary figures and images for: Long-Term Results of a Phase 2 Study of Definitive Chemoradiation Therapy Using S-1 for Esophageal Squamous Cell Carcinoma Patients Who Were Elderly or With Serious Comorbidities
Source: Front Oncol. 2022 Apr 5;12:839765. doi: 10.3389/fonc.2022.839765 (PMC9016823; doi:10.3389/fonc.2022.839765)

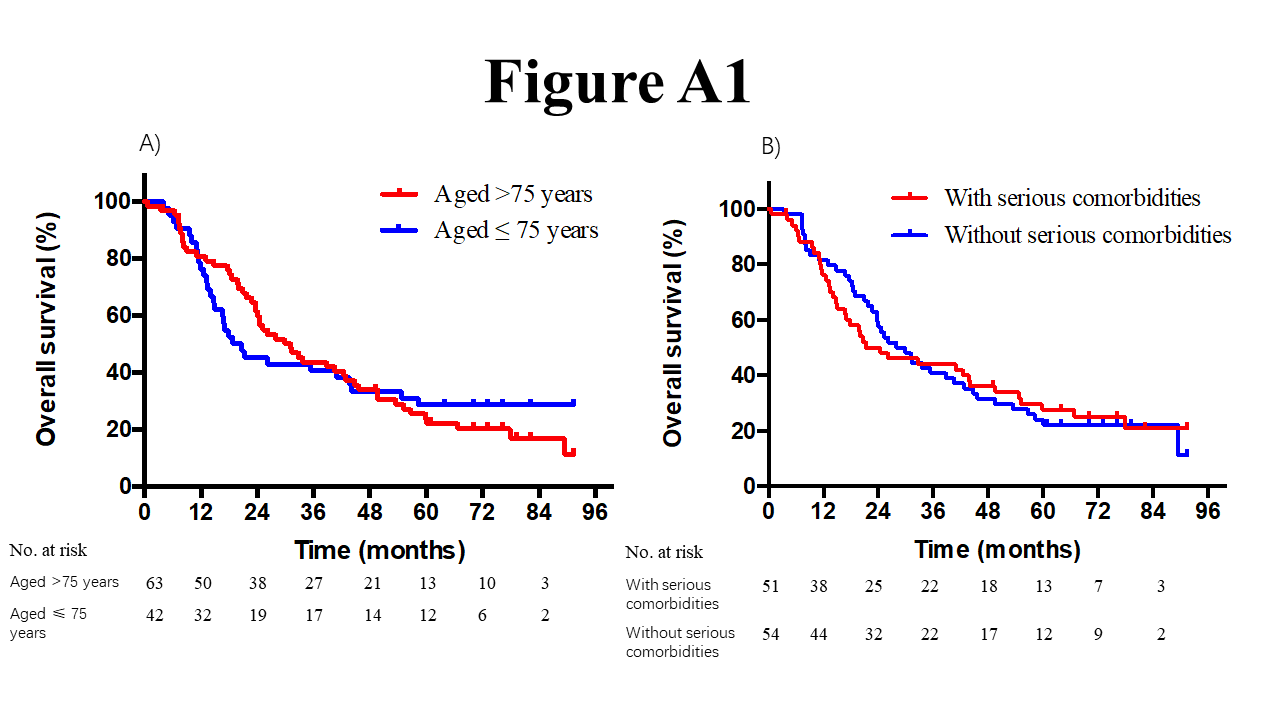

Supplement: Supplementary Figure 1 — Overall survival for subgroups of patients enrolled. No significant difference in overall survival was observed neither between the patients aged >75 years and patients aged ≤75 years (A), nor between the patients with serious comorbidities and patients without serious comorbidities (B). [file Image_1.tif]
